# Emergent SARS-CoV-2 variants: comparative replication dynamics and high sensitivity to thapsigargin

**DOI:** 10.1080/21505594.2021.2006960

**Published:** 2021-12-12

**Authors:** Sarah Al-Beltagi, Leah V. Goulding, Daniel K.E. Chang, Kenneth H. Mellits, Christopher J. Hayes, Pavel Gershkovich, Christopher M. Coleman, Kin-Chow Chang

**Affiliations:** a School of Veterinary Medicine and Science, University of Nottingham, Nottingham, UK; b The Pirbright Institute, Woking, UK; c Department of Chemical and Biological Engineering, University of Sheffield, Sheffield, UK; d School of Biosciences, University of Nottingham, Nottingham, UK; e School of Chemistry, University of Nottingham, University Park, Nottingham, UK; f School of Pharmacy, University of Nottingham, University Park, Nottingham, UK; g School of Life Sciences, University of Nottingham, University Park, Nottingham, UK

**Keywords:** SARS-CoV-2, Alpha, Beta, Delta, thapsigargin, antiviral, emergent variants, co-infection, replication synergy, syncytia

## Abstract

The struggle to control the COVID-19 pandemic is made challenging by the emergence of virulent SARS-CoV-2 variants. To gain insight into their replication dynamics, emergent Alpha (A), Beta (B) and Delta (D) SARS-CoV-2 variants were assessed for their infection performance in single variant- and co-infections. The effectiveness of thapsigargin (TG), a recently discovered broad-spectrum antiviral, against these variants was also examined. Of the 3 viruses, the D variant exhibited the highest replication rate and was most able to spread to in-contact cells; its replication rate at 24 h post-infection (hpi) based on progeny viral RNA production was over 4 times that of variant A and 9 times more than the B variant. In co-infections, the D variant boosted the replication of its co-infected partners at the expense of its own initial performance. Furthermore, co-infection with AD or AB combination conferred replication synergy where total progeny (RNA) output was greater than the sum of corresponding single-variant infections. All variants were highly sensitive to TG inhibition. A single pre-infection priming dose of TG effectively blocked all single-variant infections and every combination (AB, AD, BD variants) of co-infection at greater than 95% (relative to controls) at 72 hpi. Likewise, TG was effective in inhibiting each variant in active preexisting infection. In conclusion, against the current backdrop of the dominant D variant that could be further complicated by co-infection synergy with new variants, the growing list of viruses susceptible to TG, a promising host-centric antiviral, now includes a spectrum of contemporary SARS-CoV-2 viruses.

## Introduction

The initial success of mass vaccination against severe acute respiratory syndrome coronavirus 2 (SARS-CoV-2) to prevent or ameliorate the severe effects of coronavirus disease 2019 (COVID-19) has been marred by the continual emergence of new variants that are more infectious or virulent than the original SARS-CoV-2 isolate, and are able to reduce immune protection from existing vaccines [1]. Mutational changes of SARS-CoV-2 are actively monitored and tracked on a global scale to identify variants of concern (VOCs) that can threaten international public health. Mutations in the gene encoding spike glycoprotein (a major structural protein) and in the ORF1a (encoding non-structural proteins) account for most of the changes identified in VOCs which currently include the Alpha variant (B.1.1.7, Kent UK), Beta variant (B.1.351, South Africa), Gamma variant (P.1, Brazil) and Delta variant (B.1.617.2, India) (https://www.who.int/en/activities/tracking-SARS-CoV-2-variants/). Mutations in these variants increase virus fitness by enhancing binding to host ACE2 receptor, and/or by increasing capacity or rate of progeny virus production [2,3]. Increased expression of viral immune suppressors can also contribute to variant transmission and virulence. The rapid spread of the A variant has been attributed in part to its ability to suppress host innate immune responses from raised expression of *Orf9b* and *Orf6*, while another variant with an elongated *Orf3b* reading frame conferred greater suppression of interferon induction [4,5].

Molecular changes of SARS-CoV-2 and their associated clinical, immunological and epidemiological impact are intense areas of research. There appears, however, little published comparative information on the replication dynamics of current SARS-Cov-2 variants. There is some evidence to suggest events of recombination with SARS-CoV-2 variants in Europe and North America [6]. In one study conducted in the United Arab Emirates, at least 5% of the patients assessed were infected by more than one SARS-CoV-2 strain [7]. Here we described the replication performance of emergent SARS-CoV-2 variants to ascertain if there are competitive differences in replication rates between Alpha, Beta and Delta variants in single- and co-infections.

Recent reports on breakthrough infections in fully vaccinated individuals against SARS-CoV-2 [8,9] highlight the limitations of current vaccines against a mutating target and the need for complementary effective antivirals. We recently showed that antiviral use of thapsigargin (TG), an inhibitor of the sarcoplasmic/endoplasmic reticulum Ca^2+^ ATPase pump [10], well below cytotoxic concentrations is highly effective in blocking the replication of an early isolate of SARS-CoV-2 as well as respiratory syncytial virus (RSV), common cold coronavirus OC43, and different subtypes influenza A virus [11,12]. TG’s antiviral performance is significantly better than remdesivir and ribavirin inhibition of OC43 and RSV, respectively. Oral use of TG also protected mice against an otherwise lethal challenge of influenza virus [11,12]. Here we evaluated the sensitivity of the 3 contemporary SARS-CoV-2 variants to TG.

Of the 3 variants, we established that the D variant exhibited the highest replication rate, boosted the replication of the other variants in co-infection, and was most able to infect new cells in cell-to-cell transmission. A single pre-infection priming dose of TG effectively blocked all single-variant infections and every combination (AB, AD, BD variants) of co-infection at greater than 95% relative to controls.

## Materials and methods

### SARS-CoV-2 variants

Alpha (A or Kent) variant is a human isolate/England/202012/01B, lineage B.1.1.7; Beta (B or South Africa) variant is a human isolate/UK ex South Africa/2021, lineage B.1.351; and Delta (D or India) variant is a human isolate/UK ex-India/HCM/V/078 SARS-CoV-2, lineage B.1.617.2. All seed variants, kindly provided by UK Health Security Agency, were expanded for 3 days in Vero E6 cells grown in Opti-MEM I (Gibco) with 0.1 µg/ml TPCK-trypsin and penicillin-streptomycin (infection media) to generate stock viruses. All virus work was conducted at containment level 3 at the University of Nottingham Wolfson Centre for Global Virus Research.

### Cell culture and infection

Typically, human Calu-3 cells were primed with 0.5 µM TG (Sigma-Aldrich) or DMSO control for 30 min, washed twice with phosphate buffered saline (PBS), and infected with A variant, B variant, and D variant at 0.1 multiplicity of infection (MOI) in single variant infections (A, B and D) and in co-infections (AB, AD, and BD) for 1.5 h, washed twice with PBS and incubated in infection media. Co-infected cells received the same amount of each virus as used in single-variant infection.

### Viral RNA quantification

Viral RNA was extracted from culture media or infected cells by lysis with TRIzol reagent (Invitrogen) followed by purification using a Direct-zol RNA Miniprep kit (Zymo Research), in accordance with manufacturers’ instructions. Quantitative PCR on viral RNA isolated from culture media was performed in a Roche LightCycler using a one-step reverse-transcription-PCR kit (QuantiFast SYBR Green RT-PCR Kit, Qiagen). Annealing temperature was set at 62°C. Sequences and relative positions of primers sited on viral spike glycoprotein RNA for variant-specific qPCR are shown in Supplementary Fig. S1. Relative Ct method was used to calculate viral gene expression.

### Infective virus quantification

Focus forming assay (FFA), an immuno-cytochemical assay, was used to quantify viral spike glycoprotein-positive cells infected with spun culture supernatants. Briefly, Vero E6 cells infected with a fixed volume of supernatant for 18 h were fixed with 4% paraformaldehyde in PBS for 15 min, permeabilized with 0.1% Triton X-100 in PBS for 10 min, washed three times with PBS, followed by peroxidase treatment for 10 min and incubation with a 1:1000 dilution of primary mouse monoclonal antibody, specific to the viral spike glycoprotein (1A9 clone, GeneTex GTX632604), at room temperature for 1 h. The cells were then washed with Tris-buffered saline (TBS), incubated with horse radish peroxidase-labeled polymer for 1 h. After further washing with TBS, the cells were incubated with DAB substrate-chromogen solution for a few min (Envision+ system-HRP kit, Dako). Cells positive for viral spike glycoprotein were counted under an inverted microscope and the mean number of positive cells in six 96-wells was used to calculate infectious focus-forming units (ffu) of virus per microliter of infection volume. Visualization of cells also allows the discrimination between infected single cells and infected cell clusters.

### Quantification and statistical analysis

Statistical analysis was performed using GraphPad Prism 9 and the statistical method used is described in the figure legend. P-value <0.05 was considered significant, *p < 0.05, **p < 0.01, ***p < 0.001, ****p < 0.0001. The presented results are representative of three independent repeats, and the error bars, unless otherwise stated, are standard deviations.

## Results

### Differing replication levels and co-infection synergy of Alpha (A), Beta (B) and Delta (D) variants

**Figure 1.**
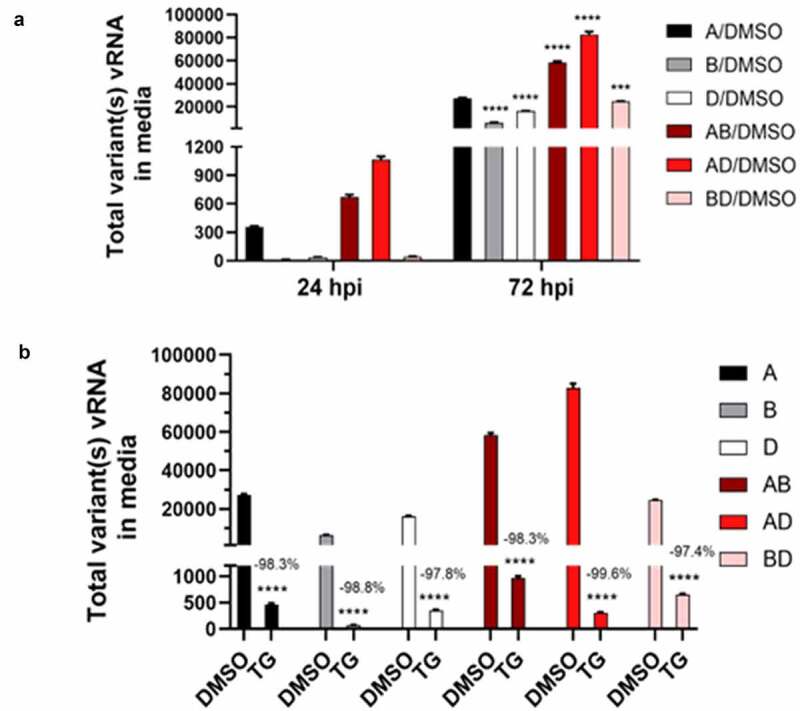
**Replication synergy in co-infection of SARS-CoV-2 variants and high antiviral potency of TG against all variants**. Confluent cells were primed with 0.5 µM TG or DMSO control for 30 min, washed twice with PBS, and infected with A variant, B variant, and D variant at 0.1 MOI in single virus infections (A, B and D) and in co-infections (AB, AD, and BD) for 1.5 h, washed twice with PBS and incubated in infection media. Notably, co-infected cells received the same amount of each virus as used in single virus infection. At 24 (panel a) and 72 hpi (panel a and b), total viral RNA from media was subjected to one-step reverse transcription qPCR, using primer set (1, 2) specific to all 3 variants, to quantify viral spike glycoprotein RNA by relative Ct method. Synergy in progeny production was evident in AB and AD co-infections where total virus RNA detected was greater than the sum of corresponding single-virus infection RNA evident at 24 and 72 hpi (panel a). Indicated significance relative to corresponding A/DMSO control based on 2-way ANOVA with Tukey’s multiple comparisons. Replication of all single virus- and co-infections was effectively blocked for at least 3 days by single pre-infection priming of TG. In AD co-infection, the most prolific infection group, combined viral RNA from TG-primed cells fell by 99.6% relative to corresponding DMSO control (panel b). Indicated significance relative to corresponding DMSO control based on 2-way ANOVA with Sidak’s multiple comparisons. Data shown are representative of three independent experiments and performed in quadruplicates

**Figure 2.**
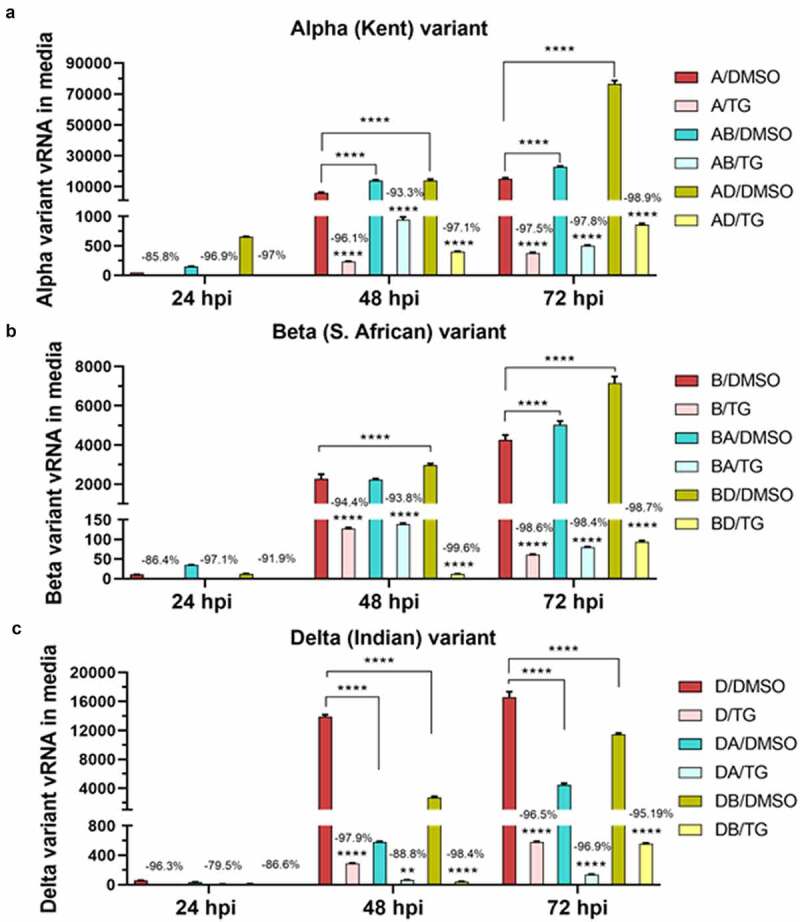
**Replication comparisons of each variant in single virus- and co-infections**. Confluent Calu-3 cells were primed with 0.5 µM TG or DMSO control for 30 min, washed twice with PBS, and infected with A variant, B variant, and D variant at 0.1 MOI in single variant infections (A, B and D) and in co-infections (AB, AD, and BD) for 1.5 h, washed twice with PBS and incubated in infection media. Co-infected cells received the same amount of each virus as in single variant infection. At 24, 48 and 72 hpi, viral RNA from media was subjected to one-step reverse transcription qPCR with variant-specific primers that can discriminate between variants in co-infected samples to detect relative spike glycoprotein gene expression. All single variant- and co-infections were blocked for at least 3 days by single pre-infection priming of TG (panels a to c). Production of A variant vRNA in AB and AD co-infections at 72 hpi was clearly enhanced (relative to single-virus A variant infection) (panel a). Production of B variant vRNA in BA and BD co-infections at 72 hpi was also enhanced (panel b). However, production of D variant vRNA in DA and DB co-infections at 72 hpi was attenuated relative to single-variant D variant infection; reduction of the D variant vRNA in DA was more than 2 times that in DB co-infection (panel c). Unless otherwise indicated significance relative to corresponding DMSO control based on 2-way ANOVA with Tukey’s multiple comparisons; % reduction is relative to corresponding DMSO group. Data shown are representative of three independent experiments and performed in quadruplicates

Calu-3 cells were highly permissive to all 3 emergent variants (A, B and D); however, production of virus progeny varied between viruses (Figure 1). Synergy in replication was evident in AD and AB co-infections where total viral RNA detected (in culture media) was greater than the sum of corresponding single-variant infections (Figure 1a). In AD co-infection, synergy was detected as early as 24 h post-infection (hpi); at 72 hpi, total viral RNA (82692 relative units [RU]) was almost two times that of the sum of separate infections (27367 + 16077 RU) (Figure 1a). RNA production of the A variant in the co-infection was more than 5 times that of its corresponding single-variant infection (76619 vs 15152 RU) (Figure 2a). The increase in A variant in AD co-infection was more than 2.5 times higher than its corresponding increase in AB co-infection (Figure 2a). However, RNA output of the D variant in the same AD co-infection was reduced by more than 70% relative to D variant only infection (from 16623 in single variant- to 4448 RU in AD co-infection) (Figure 2c). Thus, in AD co-infection, replication synergy of the A variant was at the expense of D variant performance. These data suggest that viral factors from the D variant, made in early infection, could have disproportionately benefitted *in trans* the production of its co-infected A relative.

In AB co-infection, there was also synergy in viral RNA production as early as 24 hpi (Figure 1a). Total viral RNA at 72 hpi (58260 RU) was 1.7 times the sum of corresponding single-variant infections (27367 + 6311 respectively = 33678 RU). The modest rise in A variant RNA production (from 15152 in single variant- to 22906 RU in AB co-infection) (Figure 2a) was accompanied by similar increase in B variant RNA (from 4268 in single variant to 5045 RU in AB co-infection with the A variant) (Figure 2b). Thus, in AB co-infection, viral factors from each variant could have complemented and increased the replication of the other variant.

However, in BD co-infection, total viral RNA production appeared to be additive or the sum of each participating variant (single-variant infections were 6311 + 16077 RU respectively, and BD co-infection was 24577 RU) (Figure 1a). BD co-infection improved B variant RNA production (by 2894 RU) relative to its single-variant infection (from 4268 in single B variant infection to 7162 RU in BD co-infection) (Figure 2b), but RNA production of the D variant in the same co-infection, as in AD co-infection, fell from 16623 RU in single D variant infection to 11466 RU in the co-infection (Figure 2c). Thus, in BD co-infection, the B variant was also able to benefit from and at the expense of the D variant. In summary, these data suggest that there was synergy in viral RNA production in AD and AB co-infections, and that the D variant in co-infections appeared to boost the replication of its partner.

### Replication rates of A, B and D variants in single variant- and co-infections

**Figure 3.**
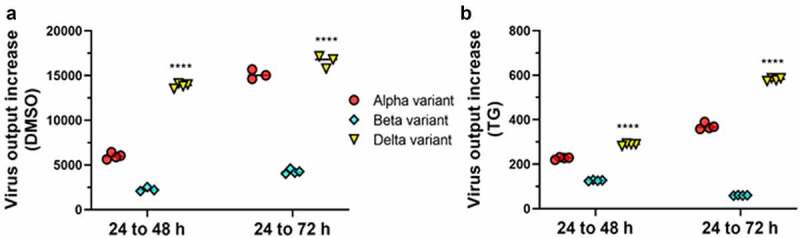
**Comparative vRNA production of emergent SARS-CoV-2 variants**. Confluent Calu-3 cells were primed with 0.5 µM TG or DMSO control for 30 min, washed twice with PBS, and separately infected with A variant, B variant, and D variant at 0.1 MOI in for 1.5 h, washed twice with PBS and incubated in infection media. At 24, 48 and 72 hpi, viral RNA from media was subjected to one-step reverse transcription qPCR with variant-specific primer sets to detect relative spike glycoprotein RNA of each variant. Increase of virus output of each variant was determined between the periods of 24 to 48 hpi, and 24 to 72 hpi. Relative rates of progeny virus production of variants are such that D > A > B (panel a). Relative increase in virus output under TG inhibition (panel b) followed the same pattern as DMSO controls but was drastically reduced. Indicated significance of D is relative to B and A variants of each corresponding interval period based on 2-way ANOVA with Tukey’s multiple comparisons. Data shown are representative of three independent experiments and performed in quadruplicates

**Figure 4.**
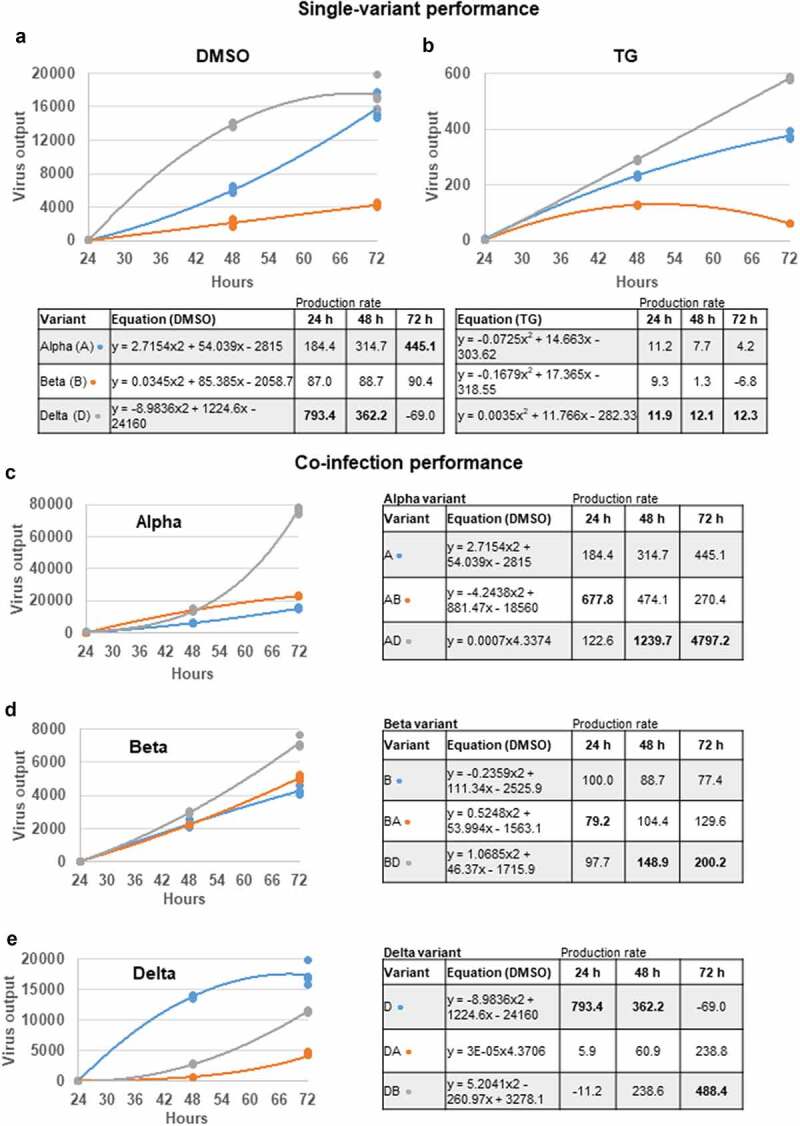
**Replication rates of A, B and D variants in single variant- and co-infections**. Data points, from earlier viral supernatant results of spike glycoprotein gene expression in single virus- and co-infections at 24, 48 and 72 hpi (Figure 2), were used to generate indicated growth curves and equations to determine the rate of viral RNA production (gradient) at a given time point. The D variant in single-variant infection, until saturation at 72 hpi, had the highest rate of viral RNA production relative to A and B variants in DMSO control (panel a) and TG-primed (panel b) Calu-3 cells. In co-infections, the D variant boosted the production rate of A and B variants (panel c and d) but at the expense of its initial performance (panel e). Negative values in production rate are likely due to virus saturation from limited cell number and even virus breakdown in media (panel a), or low initial virus growth (panel e). Data shown are representative of three independent experiments

Next, we examined the rates of replication of the 3 emergent variants. Comparisons of increase in viral RNA output of each variant, from periods of 24 to 48 hpi and 24 to 72 hpi, indicated that the D variant during early infection had the highest rate of production followed by the A and B variant in that order (Figure 3). To account for possible variation in the accuracy of MOI determination in the starting infection doses of the 3 variants, we generated a best-fit virus output curve and equation (using Microsoft Excel) from 3 data points (24, 48 and 72 hpi) of each infection group from which to calculate (dy/dx) rate of virus output at a given time point (Figure 4). In single-variant infections, the D variant indeed showed the highest replication rate at 24 hpi which then declined (most likely from limited cell supply) such that by day 3 of infection it was outperformed by the rising rate of the A variant (Figure 4a). At 24 hpi, replication rate of the D variant was more than 4 times higher (793.4/184.4) than that of variant A, and 9 times (793.4/87) more than the B variant. Variant B also showed increasing replication rate over the 3 day infection, but replication rates were persistently below those of the A variant (Figure 4a). Thus, the D variant displayed the highest replication rate from the start of infection. While virus production may fall from limited cell number in culture, the high rate of D progeny production *in vivo* would result in increased disease severity in an individual and infection rate in a population. Graphical display of replication rates of each variant (Figure 4c-e) in co-infections further highlighted the capacity of the D variant to boost the replication of co-infected A and B variants. Notably, although the replication of D variant was attenuated in early co-infection (relative to its single-variant infection), its replication rate continued to rise over the 3-day infection period, suggesting a temporary delay in rising virus production (Figure 4e).

**Figure 5.**
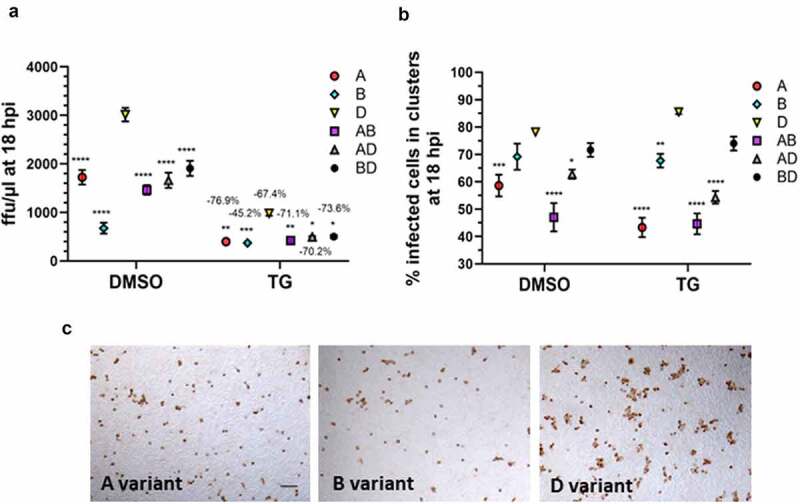
**Single-Delta variant infection exhibited the highest ability of cell-to-cell spread in 18 hpi focus forming assays (FFAs)**. Confluent Calu-3 cells were primed with 0.5 µM TG or DMSO control for 30 min, washed with PBS and infected with single variants, or combined variants in co-infections. Co-infected cells received the same amount of each virus (0.1 MOI) as in single variant infection. At 72 hpi, spun supernatants were harvested for FFAs on Vero E6 cells which were infected for 18 h followed by immunodetection of viral spike protein-positive cells to determine number of infected cells per unit volume of infecting supernatant (panel a). Despite the single Delta variant group had less starting seed virus than most other groups in the 72 hpi supernatants, after 18 h of culture in Vero E6 cells, the Delta variant produced the most number of virus positive cells per unit volume, in both DMSO and TG-primed series, indicating that it had the most rapid cell-to-cell virus spread. Indicated % reduction is relative to corresponding DMSO control (panel a). Indicated significance is relative to corresponding D variant based on 2-way ANOVA with Sidak’s multiple comparisons. Percentage of infected cells arranged in clusters of 2 or more cells indicated that the D variant was most able to form infected clusters, closely followed by mixed variants from BD co-infected (non-synergistic) cells (panel b). Indicated significance is relative to corresponding D variant based on 2-way ANOVA with Tukey’s multiple comparisons. Data shown are representative of three independent experiments. Representative FFA photomicrographs (derived from supernatants of TG-primed Calu-3 cells) show the D variant formed the largest number of clusters with 5 or more infected cells out of the three variants (panel c). Bar = 150 µM

Total viral RNA output at 72 hpi in the DMSO series was such that AD > AB > A > BD > **D** > B, and in the TG-primed series was in the order of AB > BD > A > **D** > AD > B (Figure 1b). Thus, the relative abundance of D variant RNA was at the lower end of each series. FFAs were performed in which Vero E6 cells were infected for 18 h with media of 72 hpi cells to quantify the number of infected cells by immuno-detection of viral spike glycoprotein (Figure 5). Although the starting infection titer of the D variant was smaller than those from most other infection groups, FFA showed that the D variant produced the highest number of infected cells per unit volume of supernatant (Figure 5a). FFA outcome is dictated by the starting dose of virus and the ability of the specific variant to spread to new cells by direct contact over the 18 h period (leading to syncytia formation) without prior need of extracellular virus release [13]. At least 43% of cells positive for any variant were distributed as clusters of 2 or more cells (Figure 5b). The single D variant infection had the highest percentage (>78%) of positive cells organized in clusters, relative to all other variant combinations (Figure 5b). Furthermore, the D variant was conspicuous by the abundance of large clusters of positive cells (>5 cells each) which were much less common with the other variants (Figure 5c). In summary, the D variant is superior to the A and B variants in replication rate, and is more readily able to spread to in-contact cells.

### Thapsigargin is highly effective in blocking replication of emergent SARS-CoV-2 variants in all infection combinations

**Figure 6.**
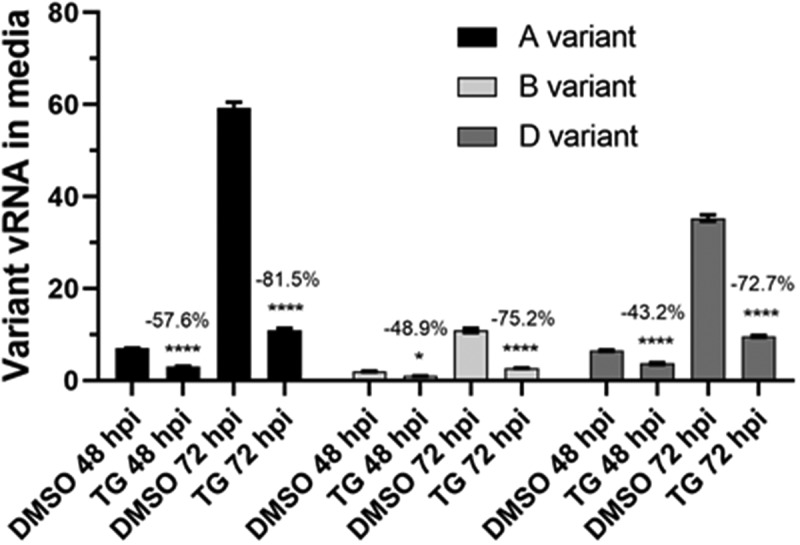
**TG was effective in inhibiting emergent SARS-CoV-2 variants in pre-infected Calu-3 cells**. Confluent cells were separately infected with SARS-CoV-2 variants at about 0.2 MOI for 2 h followed by three washes with PBS and incubated in fresh infection media. At 24 hpi, cultured media were removed and kept for re-seeding back to the same wells. Infected cells were then primed with 0.5 µM TG or DMSO control for 30 min, washed twice with PBS and the retained media were put back to the corresponding wells. At 48 and 72 hpi (i.e. at 24 h and 48 h post-priming with TG/DMSO respectively), viral RNA was extracted from spun supernatants for one-step reverse transcription qPCR to detect relative abundance of viral RNA that codes for spike glycoprotein (using primers 1 and 2). Notably, TG was able to inhibit preexisting active infection to great effect with a single 30 min exposure dose. Indicated significance relative to corresponding DMSO control based on 2-way ANOVA with Tukey’s multiple comparisons. Indicated % refers to reduction in viral detection relative to corresponding DMSO control

**Figure 7.**
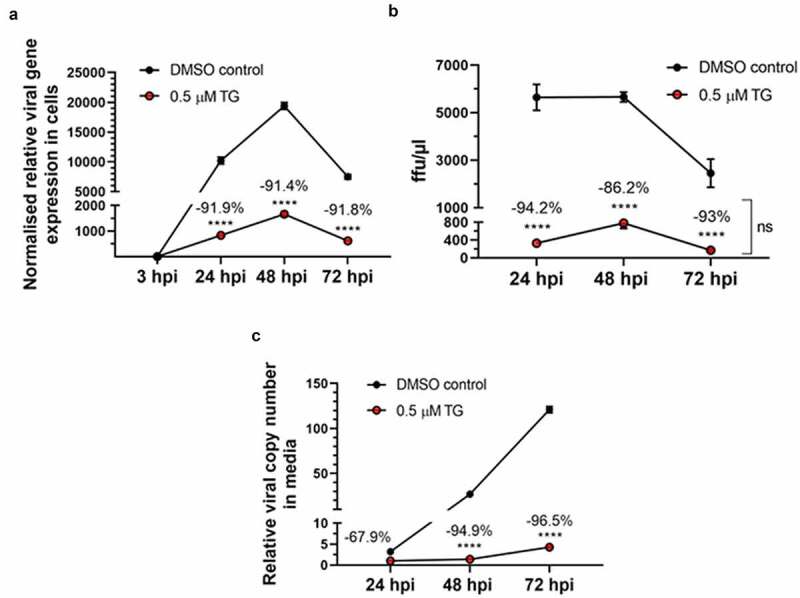
**TG reduced vRNA synthesis and progeny production of Beta variant of SARS-CoV-2 in Calu-3 cells**. Confluent cells were primed with 0.5 µM TG or DMSO control for 30 min, washed twice with PBS and infected with the B variant at 0.05 MOI in infection media for 3 h followed by three washes with PBS and incubated in fresh infection media. At indicated hpi, total cellular RNA was extracted (panel a); progeny virus in media was subjected to focus forming assay (FFA) (panel b) and viral RNA extraction (panel c). Total RNA was converted to cDNA for qPCR of SARS-CoV-2, normalized to 18s rRNA (panel a). FFA was based on the quantification of virus-positive Vero E6 cells at 18 hpi by immunochemical detection of viral spike glycoprotein (error bars = SEM; ns = not significant between time points) (panel b). Viral RNA from media was subjected to one-step reverse transcription qPCR to detect relative copy number of SARS-CoV-2, based on relative Ct method (panel c). Notably, despite increasing viral RNA accumulation in media of infected cells with time (panel c), viral gene expression (panel a) and infectious progeny virus (panel b) were in relative decline by 72 hpi. Indicated significance relative to corresponding DMSO control based on 2-way ANOVA with Sidak’s multiple comparisons. Indicated % refers to reduction in viral detection relative to corresponding DMSO control

The antiviral use of TG against coronavirus, influenza virus and respiratory syncytial virus has recently been shown to be non-cytotoxic in a variety of human cells, including primary NHBE cells and A549 cells [11,12]. We found that the antiviral doses of TG to prime Calu-3 cells also had little or no effect on cell viability based on luminescence ATP and MT assays (Supplementary Fig. S2). SARS-CoV-2 replication in all single variant- and co-infection combinations was effectively blocked for at least 3 days by a single 30 min pre-infection priming of TG (Figures 1b, 2, 3b and 4b). At 72 hpi, there was greater than 95% reduction in total (Figure 1b) or variant-specific (Figure 2) progeny virus RNA output. In AD co-infection, the most prolific infection group, combined viral RNA from TG-primed cells fell by 99.6% relative to corresponding DMSO control (Figure 1b). Although percentage reduction in infectious progeny production from TG primed cells at 72 hpi, as determined by FFA (Figure 5a), was not as dramatic as in viral RNA reduction (Figures 1b and 2), viable progeny virus in all infection combinations was reduced by at least 45% relative to corresponding DMSO control (Figure 5a). As intimated earlier, such quantitative differences in % viral reduction by TG between progeny viral RNA (Figures 1b and 2) and corresponding infectious virions (Figure 5a) were due in part to new infection of bystander cells in the latter during the 18 h FFA period, and other experimental variables such as reduction in virus viability from stagnation in the 3-day infection culture (Figure 7b). We further showed that TG was effective in inhibiting the replication of the three variants during active infection (Figure 6). Progeny vRNA detected from Calu-3 cells, pre-infected with each variant for 24 h, was drastically reduced following TG priming for 30 min, suggesting its potential therapeutic value post-virus entry (Figure 6). TG inhibited SARS-CoV-2 replication, as shown by reduced RNA synthesis (Figure 7a) with corresponding reduction in progeny virus production (Figure 7b,c). In summary, TG as an antiviral is highly effective against the A, B and D variants of SARS-CoV-2 in all combinations of infection.

## Discussion

A key finding from single-variant comparisons of SARS-CoV-2 infection is that the D variant is superior to the A and B variants in replication rate and in cell-to-cell transmission. Technically, the use of FFA in virus quantification, but not the use of median tissue culture infectious dose (TCID_50_) assay [11,14], has an added advantage of visualizing and quantifying infected cell clusters from direct collateral spread of SARS-CoV-2. Our *in vitro* finding of high replication rate of the D variant is consistent with a recent finding that in nasopharyngeal samples, virus load of the Delta variant was 2.5-fold higher (*p* < 0.05) than that of the Beta variant [15]; and with a pre-print report based on clinical cases that found the D variant to proliferate more rapidly and accumulate to much higher levels (~100 times higher) in the respiratory tract than the first wave of 2020 variants [16].

This work has also highlighted that co-infection in certain SARS-CoV-2 variant combinations, such as the AD or AB pairing, can result in replication synergy. AD co-infection was particularly striking in that the A variant was quantitatively dominant over and at the expense of the D variant in progeny RNA production. Although there was no significant change in total infectious progeny, as detected by 18 h FFAs, between A variant single-infection and AD co-infection, we should closely monitor events of co-infection, in particular of new variants, for disease severity and population spread as the dynamics of virus replication in co-infection are unpredictable and can differ sharply from corresponding single-virus infections. In a case study, co-infection by two SARS-CoV-2 lineages, 20A and 20B, was thought to contribute to the extended duration and severity of disease in a 17-year-old patient [17].

The worrying breakthrough rate of D variant infection amongst fully vaccinated individuals suggests that current vaccines are less able to block virus proliferation to prevent transmission. The rapid replication rate and cell-to-cell spread of the D variant are likely viral traits responsible for infection breakthrough in fully vaccinated individuals. The ability of the D variant to rapidly spread through cells by direct contact without the prior need of extracellular progeny release could partly shield the virus from an existing humoral response. To tackle disease fallout from increased virus pathogenicity, infectivity and replication synergy, future management of COVID-19 may well require the use of contemporary multivalent vaccines, combined with effective broad-spectrum antivirals that can preferably be administered orally. We previously showed that the antiviral use of TG was highly effective against influenza viruses of different subtypes [12], respiratory syncytial virus, coronavirus OC43 and an original isolate of SARS-CoV-2 (2019-nCoV/Italy-INMI1, clade V) [11]. Other groups have reported effective TG inhibition of paramyxoviruses [18], and 229E, Middle-East respiratory syndrome and SARS-CoV-2 coronaviruses [19]. All available data (generated by us and others) as exemplified in influenza virus, RSV and coronaviruses, including SARS-CoV-2, indicate that TG does not prevent viral entry but rather triggers intracellular pathways to inhibit virus replication [11,12,19]. As a host-centric antiviral, TG hits several central host mechanisms connected to endoplasmic reticulum stress-unfolded protein response to inhibit several stages of virus replication. The antiviral potency of TG has now been extended to contemporary SARS-CoV-2 variants, including the D variant, in all combinations of single- and co-infections. We therefore submit that TG is potentially a truly broad-spectrum antiviral that targets a growing list of viruses.

## Supplementary Material

Supplemental Material

## Data Availability

All primary data from which all results are based and as presented in the figures are available from the corresponding author upon reasonable request.
