# Supplementary figures and images for: Emergent SARS-CoV-2 variants: comparative replication dynamics and high sensitivity to thapsigargin
Source: Virulence. 2021 Dec 12;12(1):2946–56. doi: 10.1080/21505594.2021.2006960 (PMC8667886; doi:10.1080/21505594.2021.2006960)

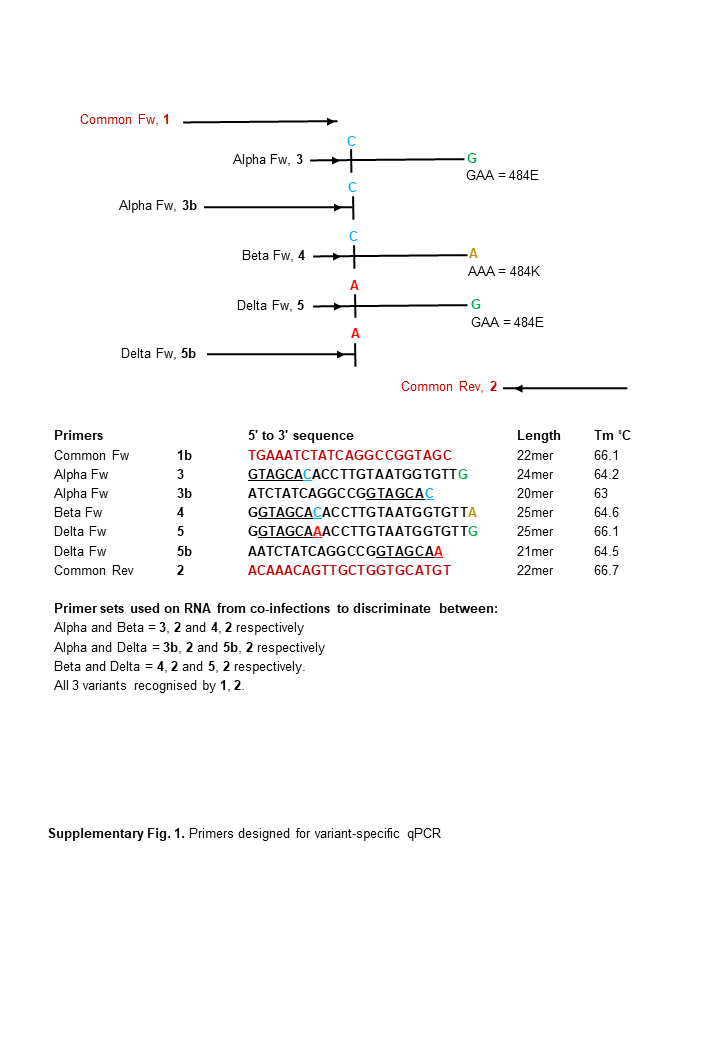

Supplement: Supplemental Material [file KVIR_A_2006960_SM0866.zip › supplementary/Supplementary_Figure_1.tif]

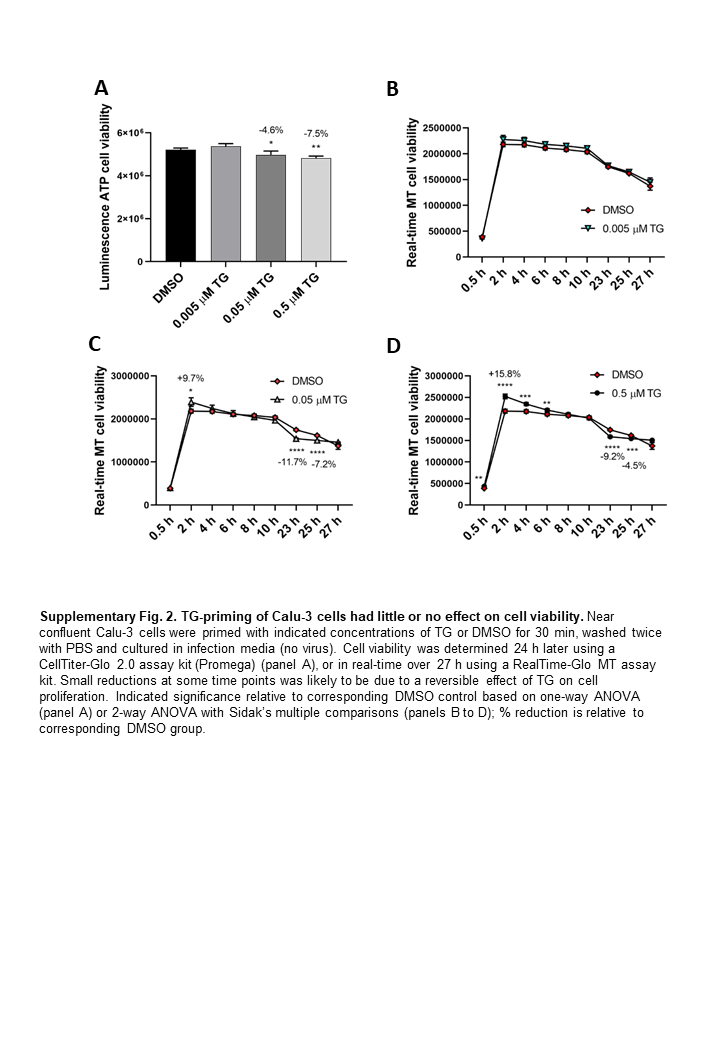

Supplement: Supplemental Material [file KVIR_A_2006960_SM0866.zip › supplementary/Supplementary_Figure_2.tif]
